# Supplementary material for: Pyruvate Dehydrogenase Kinase 1 inhibition mediated oxidative phosphorylation enhancement in cartilage promotes osteoarthritis progression
Source: BMC Musculoskelet Disord. 2023 Jul 20;24:597. doi: 10.1186/s12891-023-06585-6 (PMC10357736; doi:10.1186/s12891-023-06585-6)
Supplement: Supplementary file 1 — Additional file 1. [file 12891_2023_6585_MOESM1_ESM.zip › BP.Gsea.1653623667859/GOBP_EMBRYONIC_PLACENTA_DEVELOPMENT.html]

Details for gene set GOBP\_EMBRYONIC\_PLACENTA\_DEVELOPMENT[GSEA]

|  || Dataset | 55457\_collapsed\_to\_symbols.cls.cls #OA\_versus\_normal.cls.cls #OA\_versus\_normal\_repos |
| Phenotype | cls.cls#OA\_versus\_normal\_repos |
| Upregulated in class | normal |
| GeneSet | GOBP\_EMBRYONIC\_PLACENTA\_DEVELOPMENT |
| Enrichment Score (ES) | -0.44429365 |
| Normalized Enrichment Score (NES) | -1.8586918 |
| Nominal p-value | 0.0 |
| FDR q-value | 0.06568557 |
| FWER p-Value | 0.868 |
Table: GSEA Results Summary

  

Fig 1: Enrichment plot: GOBP\_EMBRYONIC\_PLACENTA\_DEVELOPMENT      
 Profile of the Running ES Score & Positions of GeneSet Members on the Rank Ordered List

  

| SYMBOL | TITLE | RANK IN GENE LIST | RANK METRIC SCORE | RUNNING ES | CORE ENRICHMENT || 1 | SPINT2 | "serine peptidase inhibitor, Kunitz type 2 [Source:HGNC Symbol;Acc:HGNC:11247]" | 541 | 0.645 | -0.0167 | No |
| 2 | AKT1 | AKT serine/threonine kinase 1 [Source:HGNC Symbol;Acc:HGNC:391] | 1192 | 0.478 | -0.0485 | No |
| 3 | CEBPA | CCAAT enhancer binding protein alpha [Source:HGNC Symbol;Acc:HGNC:1833] | 1750 | 0.392 | -0.0765 | No |
| 4 | GRHL2 | grainyhead like transcription factor 2 [Source:HGNC Symbol;Acc:HGNC:2799] | 2111 | 0.348 | -0.0908 | No |
| 5 | KRT19 | keratin 19 [Source:HGNC Symbol;Acc:HGNC:6436] | 2258 | 0.331 | -0.0891 | No |
| 6 | WNT2 | Wnt family member 2 [Source:HGNC Symbol;Acc:HGNC:12780] | 2700 | 0.279 | -0.1125 | No |
| 7 | SOX15 | SRY-box transcription factor 15 [Source:HGNC Symbol;Acc:HGNC:11196] | 2748 | 0.275 | -0.1053 | No |
| 8 | SENP2 | SUMO specific peptidase 2 [Source:HGNC Symbol;Acc:HGNC:23116] | 3040 | 0.246 | -0.1183 | No |
| 9 | PCDH12 | protocadherin 12 [Source:HGNC Symbol;Acc:HGNC:8657] | 3081 | 0.242 | -0.1118 | No |
| 10 | TFEB | transcription factor EB [Source:HGNC Symbol;Acc:HGNC:11753] | 3190 | 0.232 | -0.1111 | No |
| 11 | TRIM28 | tripartite motif containing 28 [Source:HGNC Symbol;Acc:HGNC:16384] | 3526 | 0.200 | -0.1293 | No |
| 12 | CSF2 | colony stimulating factor 2 [Source:HGNC Symbol;Acc:HGNC:2434] | 3699 | 0.185 | -0.1354 | No |
| 13 | ST14 | ST14 transmembrane serine protease matriptase [Source:HGNC Symbol;Acc:HGNC:11344] | 3900 | 0.165 | -0.1445 | No |
| 14 | SPINT1 | "serine peptidase inhibitor, Kunitz type 1 [Source:HGNC Symbol;Acc:HGNC:11246]" | 3938 | 0.161 | -0.1410 | No |
| 15 | HEY2 | hes related family bHLH transcription factor with YRPW motif 2 [Source:HGNC Symbol;Acc:HGNC:4881] | 4058 | 0.150 | -0.1444 | No |
| 16 | CDKN1C | cyclin dependent kinase inhibitor 1C [Source:HGNC Symbol;Acc:HGNC:1786] | 4233 | 0.134 | -0.1527 | No |
| 17 | VASH2 | vasohibin 2 [Source:HGNC Symbol;Acc:HGNC:25723] | 4345 | 0.124 | -0.1564 | No |
| 18 | IGF2 | insulin like growth factor 2 [Source:HGNC Symbol;Acc:HGNC:5466] | 4378 | 0.123 | -0.1541 | No |
| 19 | OVOL2 | ovo like zinc finger 2 [Source:HGNC Symbol;Acc:HGNC:15804] | 5017 | 0.071 | -0.2010 | No |
| 20 | ASCL2 | achaete-scute family bHLH transcription factor 2 [Source:HGNC Symbol;Acc:HGNC:739] | 5194 | 0.057 | -0.2125 | No |
| 21 | FZD5 | frizzled class receptor 5 [Source:HGNC Symbol;Acc:HGNC:4043] | 5392 | 0.041 | -0.2262 | No |
| 22 | E2F8 | E2F transcription factor 8 [Source:HGNC Symbol;Acc:HGNC:24727] | 5527 | 0.031 | -0.2355 | No |
| 23 | GJB5 | gap junction protein beta 5 [Source:HGNC Symbol;Acc:HGNC:4287] | 5542 | 0.029 | -0.2354 | No |
| 24 | GRB2 | growth factor receptor bound protein 2 [Source:HGNC Symbol;Acc:HGNC:4566] | 5697 | 0.016 | -0.2468 | No |
| 25 | TMED2 | transmembrane p24 trafficking protein 2 [Source:HGNC Symbol;Acc:HGNC:16996] | 5709 | 0.015 | -0.2471 | No |
| 26 | PLK4 | polo like kinase 4 [Source:HGNC Symbol;Acc:HGNC:11397] | 6041 | -0.008 | -0.2726 | No |
| 27 | WNT7B | Wnt family member 7B [Source:HGNC Symbol;Acc:HGNC:12787] | 6230 | -0.023 | -0.2863 | No |
| 28 | PKD2 | "polycystin 2, transient receptor potential cation channel [Source:HGNC Symbol;Acc:HGNC:9009]" | 6254 | -0.024 | -0.2872 | No |
| 29 | GGNBP2 | gametogenetin binding protein 2 [Source:HGNC Symbol;Acc:HGNC:19357] | 6301 | -0.027 | -0.2897 | No |
| 30 | TTPA | alpha tocopherol transfer protein [Source:HGNC Symbol;Acc:HGNC:12404] | 6796 | -0.063 | -0.3257 | No |
| 31 | MAPK1 | mitogen-activated protein kinase 1 [Source:HGNC Symbol;Acc:HGNC:6871] | 6853 | -0.068 | -0.3274 | No |
| 32 | SETD2 | "SET domain containing 2, histone lysine methyltransferase [Source:HGNC Symbol;Acc:HGNC:18420]" | 6926 | -0.074 | -0.3301 | No |
| 33 | SP3 | Sp3 transcription factor [Source:HGNC Symbol;Acc:HGNC:11208] | 7033 | -0.082 | -0.3351 | No |
| 34 | CITED2 | Cbp/p300 interacting transactivator with Glu/Asp rich carboxy-terminal domain 2 [Source:HGNC Symbol;Acc:HGNC:1987] | 7100 | -0.087 | -0.3368 | No |
| 35 | FGFR2 | fibroblast growth factor receptor 2 [Source:HGNC Symbol;Acc:HGNC:3689] | 7374 | -0.108 | -0.3538 | No |
| 36 | LEF1 | lymphoid enhancer binding factor 1 [Source:HGNC Symbol;Acc:HGNC:6551] | 7467 | -0.116 | -0.3565 | No |
| 37 | CDX4 | caudal type homeobox 4 [Source:HGNC Symbol;Acc:HGNC:1808] | 7519 | -0.120 | -0.3557 | No |
| 38 | HEY1 | hes related family bHLH transcription factor with YRPW motif 1 [Source:HGNC Symbol;Acc:HGNC:4880] | 8213 | -0.172 | -0.4029 | No |
| 39 | VASH1 | vasohibin 1 [Source:HGNC Symbol;Acc:HGNC:19964] | 8473 | -0.191 | -0.4156 | No |
| 40 | BMP5 | bone morphogenetic protein 5 [Source:HGNC Symbol;Acc:HGNC:1072] | 8488 | -0.193 | -0.4090 | No |
| 41 | PKD1 | "polycystin 1, transient receptor potential channel interacting [Source:HGNC Symbol;Acc:HGNC:9008]" | 8755 | -0.213 | -0.4214 | No |
| 42 | RBPJ | recombination signal binding protein for immunoglobulin kappa J region [Source:HGNC Symbol;Acc:HGNC:5724] | 8800 | -0.217 | -0.4163 | No |
| 43 | IL10 | interleukin 10 [Source:HGNC Symbol;Acc:HGNC:5962] | 8855 | -0.221 | -0.4117 | No |
| 44 | PHLDA2 | pleckstrin homology like domain family A member 2 [Source:HGNC Symbol;Acc:HGNC:12385] | 9059 | -0.237 | -0.4182 | No |
| 45 | HAND1 | heart and neural crest derivatives expressed 1 [Source:HGNC Symbol;Acc:HGNC:4807] | 9082 | -0.238 | -0.4105 | No |
| 46 | NCOA1 | nuclear receptor coactivator 1 [Source:HGNC Symbol;Acc:HGNC:7668] | 9270 | -0.253 | -0.4151 | No |
| 47 | ARNT | aryl hydrocarbon receptor nuclear translocator [Source:HGNC Symbol;Acc:HGNC:700] | 9427 | -0.267 | -0.4167 | No |
| 48 | BMP7 | bone morphogenetic protein 7 [Source:HGNC Symbol;Acc:HGNC:1074] | 9544 | -0.278 | -0.4148 | No |
| 49 | MDFI | MyoD family inhibitor [Source:HGNC Symbol;Acc:HGNC:6967] | 9923 | -0.314 | -0.4319 | Yes |
| 50 | CDX2 | caudal type homeobox 2 [Source:HGNC Symbol;Acc:HGNC:1806] | 10078 | -0.328 | -0.4310 | Yes |
| 51 | NR2F2 | nuclear receptor subfamily 2 group F member 2 [Source:HGNC Symbol;Acc:HGNC:7976] | 10129 | -0.334 | -0.4217 | Yes |
| 52 | MAP2K1 | mitogen-activated protein kinase kinase 1 [Source:HGNC Symbol;Acc:HGNC:6840] | 10197 | -0.340 | -0.4135 | Yes |
| 53 | GCM1 | glial cells missing transcription factor 1 [Source:HGNC Symbol;Acc:HGNC:4197] | 10236 | -0.345 | -0.4028 | Yes |
| 54 | PRDM1 | PR/SET domain 1 [Source:HGNC Symbol;Acc:HGNC:9346] | 10374 | -0.358 | -0.3993 | Yes |
| 55 | CASP8 | caspase 8 [Source:HGNC Symbol;Acc:HGNC:1509] | 10442 | -0.366 | -0.3901 | Yes |
| 56 | STK3 | serine/threonine kinase 3 [Source:HGNC Symbol;Acc:HGNC:11406] | 10656 | -0.388 | -0.3914 | Yes |
| 57 | DNAJB6 | DnaJ heat shock protein family (Hsp40) member B6 [Source:HGNC Symbol;Acc:HGNC:14888] | 10702 | -0.394 | -0.3793 | Yes |
| 58 | PLG | plasminogen [Source:HGNC Symbol;Acc:HGNC:9071] | 10939 | -0.423 | -0.3810 | Yes |
| 59 | GATA2 | GATA binding protein 2 [Source:HGNC Symbol;Acc:HGNC:4171] | 11058 | -0.439 | -0.3729 | Yes |
| 60 | STK4 | serine/threonine kinase 4 [Source:HGNC Symbol;Acc:HGNC:11408] | 11074 | -0.441 | -0.3567 | Yes |
| 61 | MED1 | mediator complex subunit 1 [Source:HGNC Symbol;Acc:HGNC:9234] | 11146 | -0.450 | -0.3445 | Yes |
| 62 | ADM | adrenomedullin [Source:HGNC Symbol;Acc:HGNC:259] | 11543 | -0.506 | -0.3553 | Yes |
| 63 | NSDHL | NAD(P) dependent steroid dehydrogenase-like [Source:HGNC Symbol;Acc:HGNC:13398] | 11670 | -0.527 | -0.3444 | Yes |
| 64 | EPAS1 | endothelial PAS domain protein 1 [Source:HGNC Symbol;Acc:HGNC:3374] | 11758 | -0.542 | -0.3297 | Yes |
| 65 | HIF1A | hypoxia inducible factor 1 subunit alpha [Source:HGNC Symbol;Acc:HGNC:4910] | 11833 | -0.556 | -0.3136 | Yes |
| 66 | BPTF | bromodomain PHD finger transcription factor [Source:HGNC Symbol;Acc:HGNC:3581] | 11870 | -0.565 | -0.2941 | Yes |
| 67 | HS6ST1 | heparan sulfate 6-O-sulfotransferase 1 [Source:HGNC Symbol;Acc:HGNC:5201] | 11932 | -0.579 | -0.2760 | Yes |
| 68 | LIF | LIF interleukin 6 family cytokine [Source:HGNC Symbol;Acc:HGNC:6596] | 12085 | -0.615 | -0.2635 | Yes |
| 69 | EGFR | epidermal growth factor receptor [Source:HGNC Symbol;Acc:HGNC:3236] | 12109 | -0.622 | -0.2407 | Yes |
| 70 | SNAI1 | snail family transcriptional repressor 1 [Source:HGNC Symbol;Acc:HGNC:11128] | 12365 | -0.696 | -0.2331 | Yes |
| 71 | PDGFB | platelet derived growth factor subunit B [Source:HGNC Symbol;Acc:HGNC:8800] | 12389 | -0.704 | -0.2071 | Yes |
| 72 | SOCS3 | suppressor of cytokine signaling 3 [Source:HGNC Symbol;Acc:HGNC:19391] | 12453 | -0.725 | -0.1834 | Yes |
| 73 | JUNB | "JunB proto-oncogene, AP-1 transcription factor subunit [Source:HGNC Symbol;Acc:HGNC:6205]" | 12468 | -0.734 | -0.1555 | Yes |
| 74 | CITED1 | Cbp/p300 interacting transactivator with Glu/Asp rich carboxy-terminal domain 1 [Source:HGNC Symbol;Acc:HGNC:1986] | 12493 | -0.746 | -0.1279 | Yes |
| 75 | HES1 | hes family bHLH transcription factor 1 [Source:HGNC Symbol;Acc:HGNC:5192] | 12676 | -0.849 | -0.1086 | Yes |
| 76 | CCN1 | cellular communication network factor 1 [Source:HGNC Symbol;Acc:HGNC:2654] | 12802 | -0.970 | -0.0801 | Yes |
| 77 | CEBPB | CCAAT enhancer binding protein beta [Source:HGNC Symbol;Acc:HGNC:1834] | 12804 | -0.973 | -0.0417 | Yes |
| 78 | ZFP36L1 | ZFP36 ring finger protein like 1 [Source:HGNC Symbol;Acc:HGNC:1107] | 12888 | -1.244 | 0.0009 | Yes |
Table: GSEA details [plain text format]

  

Fig 2: GOBP\_EMBRYONIC\_PLACENTA\_DEVELOPMENT      
 Blue-Pink O' Gram in the Space of the Analyzed GeneSet

  

Fig 3: GOBP\_EMBRYONIC\_PLACENTA\_DEVELOPMENT: Random ES distribution      
 Gene set null distribution of ES for **GOBP\_EMBRYONIC\_PLACENTA\_DEVELOPMENT**
